# Supplementary material for: Metabolic regulation of subcellular sucrose cleavage inferred from quantitative analysis of metabolic functions
Source: Quant Plant Biol. 2022 Jun 13;3:e10. doi: 10.1017/qpb.2022.5 (PMC10095975; doi:10.1017/qpb.2022.5)
Supplement: Supplementary file 1 [file S2632882822000054sup001.pdf]

Supplement

Deriving a Jacobian matrix to study invertase-catalysed sucrose hydrolysis

Due to its central role in sugar metabolism, a quantitative understanding of invertase kinetics is crucial for analysis and interpretation of photosynthesis and plant carbohydrate metabolism. Also, sugar signalling, plant fertility and fitness are significantly affected and regulated by invertases which emphasises even more their essential role in plants (Wan et al., 2018). Yet, as soon as enzyme kinetics recorded *in vitro* are applied to explain *in vivo* metabolic regulation and to simulate metabolic fluxes, further information about involved metabolite pools, compartments and enzyme regulation is needed in order to estimate dynamics of substrate (*here*: sucrose) and product (*here*: hexose) concentrations. If metabolite concentrations and kinetic parameters are known, the system of ODEs which describes dynamics of sucrose and hexose concentrations can be numerically solved, i.e., numerically integrated. Thus, to estimate the impact of product inhibition of invertases, dynamics of hexoses need to be estimated which depend on invertase activity (input) and output reactions which consume or interconvert glucose and fructose (here: $r_{out,Glc}$ and $r_{out,Frc}$). A physiologically important output reaction, which interconverts hexoses, is their phosphorylation, catalysed by hexokinases (Granot et al., 2014). To prevent the depletion of sucrose, an input function needs to be defined which supplies the system with sucrose molecules (r_{in}). In plant metabolism, sucrose phosphate synthase (SPS) catalyses and regulates sucrose biosynthesis in the cytosol together with fructose-1,6-bisphosphatase (FBPase) (Doehlert & Huber, 1983; Stitt et al., 1983; Volkert et al., 2014). Here, the input reaction rate r_{in} was defined to be constant without explicitly modelling SPS or FBPase kinetics (Eq. S1). Invertase reaction and hexose output followed Michaelis-Menten kinetics considering product inhibition of invertase (Eq. S2; Kitashova et al., 2021) while output was assumed not to be inhibited (Eqs. S3, S4).

$$r_{in} = constant$$

(Eq. S1)

$$r_{Inv} = \frac{V_{max,inv}\, Suc}{\left(K_{M,Suc}\left(1 + {Frc}/{K_{i,Frc}}\right) + Suc\right)\left(1 + {Glc}/{K_{i,Glc}}\right)}$$

(Eq. S2)

$$r_{out,Glc} = \frac{V_{max,out,Glc}\, Glc}{\left(K_{M,out,Glc} + Glc\right)}$$

$$(\mathrm{Eq. S3})$$

$$r_{out,Frc} = \frac{V_{max,out,Frc}\, Frc}{\left(K_{M,out,Frc} + Frc\right)}$$

$$(\mathrm{Eq. S4})$$

Parameters $V_{max,...}$ are the maximum velocities of enzyme reactions, i.e., reaction rates under substrate saturation. Michaelis constants $K_{M,...}$ are affinities of enzymes for their substrates and represent the metabolite concentration at r = $V_{max}/2$. The inhibitory constants $K_{i,...}$ indicate inhibitor concentrations which are needed to reduce enzyme velocity to half maximum. To study complex system properties like stabilization after perturbation, biochemical systems are typically linearised and considered near a steady state. Yet, due to strong external and internal dynamics, plant metabolism can hardly be described by steady state assumptions, which is d**M(t)**/dt = **0** where **M(t)** represents a vector of metabolite concentrations and **0** is the zero vector. In the given example of sucrose hydrolysis within plant cells, both substrate and product molecules may show significant dynamics within a diurnal cycle (Sulpice et al., 2014). Although this clearly constrains the interpretation of findings made by assumptions of a steady state, it simplifies theoretical and computational analysis of metabolic systems.

Additionally, analytical solutions of enzymatic reaction systems can be obtained using the assumption that velocity of an enzymatic reaction linearly depends on substrate concentrations (Heinrich & Rapoport, 1974). In contrast, solving nonlinear metabolic systems analytically is hardly possible. Taylor expansion of temporal changes of deviations from a steady state leads to the Jacobian matrix of a reaction system (Klipp et al., 2016). In context of metabolic networks, the Jacobian matrix describes elasticities of metabolic functions towards dynamics of metabolite concentrations. For the given reaction system (Eq. 2, main text), the Jacobian matrix reads (Eq. S5):

$$\boldsymbol{J} = \begin{pmatrix} j_{11} & j_{12} & j_{13} \\ j_{21} & j_{22} & j_{23} \\ j_{31} & j_{32} & j_{33} \end{pmatrix} = \begin{pmatrix} \frac{\partial(f(Suc))}{\partial(Suc)} & \frac{\partial(f(Suc))}{\partial(Glc)} & \frac{\partial(f(Suc))}{\partial(Frc)} \\ \frac{\partial(f(Glc))}{\partial(Suc)} & \frac{\partial(f(Glc))}{\partial(Glc)} & \frac{\partial(f(Glc))}{\partial(Frc)} \\ \frac{\partial(f(Frc))}{\partial(Suc)} & \frac{\partial(f(Frc))}{\partial(Glc)} & \frac{\partial(f(Frc))}{\partial(Frc)} \end{pmatrix}$$

$$= \begin{pmatrix} \frac{\partial(r_{in} - r_{inv})}{\partial(Suc)} & \frac{\partial(r_{in} - r_{inv})}{\partial(Glc)} & \frac{\partial(r_{in} - r_{inv})}{\partial(Frc)} \\ \frac{\partial(r_{Inv} - r_{out,Glc})}{\partial(Suc)} & \frac{\partial(r_{Inv} - r_{out,Glc})}{\partial(Glc)} & \frac{\partial(r_{Inv} - r_{out,Glc})}{\partial(Frc)} \\ \frac{\partial(r_{Inv} - r_{out,Frc})}{\partial(Suc)} & \frac{\partial(r_{Inv} - r_{out,Frc})}{\partial(Glc)} & \frac{\partial(r_{Inv} - r_{out,Frc})}{\partial(Frc)} \end{pmatrix}$$

(Eq. S5)

with diagonal entries (Eqs. S6-S8),

$$\frac{\partial(r_{in} - r_{inv})}{\partial(Suc)} = -\frac{K_{M,Suc}K_{i,Glc}K_{i,Frc}V_{max,inv}(Frc + K_{i,Frc})}{(Glc + K_{i,Glc})(K_{M,Suc}\,Frc + K_{M,Suc}\,K_{i,Frc} + K_{i,Frc}Suc)^2}$$

(Eq. S6)

$$\frac{\partial(r_{Inv} - r_{out,Glc})}{\partial(Glc)} = -\frac{K_{i,Frc}K_{i,Glc}V_{max,inv}\,Suc}{(Glc + K_{i,Glc})^2(K_{M,Suc}\,Frc + K_{M,Suc}\,K_{i,Frc} + K_{i,Frc}\,Suc)} - \frac{V_{max,out,Glc}K_{M,out,Glc}}{(K_{M,out,Glc} + Glc)^2}$$

(Eq. S7)

$$\frac{\partial(r_{Inv} - r_{out,Frc})}{\partial(Frc)} = -\frac{K_{M,Suc}\,K_{i,Frc}K_{i,Glc}V_{max,inv}\,Suc}{(Glc + K_{i,Glc})(K_{M,Suc}\,Frc + K_{M,Suc}K_{i,Frc} + K_{i,Frc}Suc)^2} - \frac{V_{max,out,Frc}K_{M,out,Frc}}{(K_{M,out,Frc} + Frc)^2}$$

(Eq. S8)

In words, the diagonal entries represent elasticities of functions of metabolites with regard to dynamics in their own concentration. This reflects that metabolites are affecting their own concentration dynamics by participating as substrates and/or products in enzymatic reactions. Non-diagonal entries of ***J*** reflect any regulatory effects of metabolites on all other metabolic functions within a reaction system (equations of derivatives are not explicitly shown). Further, the equations show that, depending on the degree of substrate saturation of an enzyme, dynamics of substrate concentrations have a differential effect on the Jacobian trajectories. For example, if enzymes, catalysing the $r_{out,Glc}$ and $r_{out,Frc}$ reactions, are fully saturated, further

increase of substrate concentration (here: Glc and Frc) results in exponential approximation towards zero of the second term in Eqs. S7 and S8. Hence, under such conditions where reaction products of invertases strongly accumulate, hexose consuming reactions have a lower impact on hexose dynamics than under non-saturated conditions. At the same time, (strong) hexose accumulation results in invertase inhibition which limits invertase-induced sucrose dynamics because (Eq. S9):

$$\text{if Glc >> Suc and Frc >> Suc, then } \lim_{Glc,Frc \to \infty} \frac{\partial(r_{in} - r_{inv})}{\partial(Suc)} = 0 \quad \text{(Eq. S9)}$$

These considerations are simplified because also enzyme parameters need to be considered in order to determine the Jacobian entries as well as their dynamics under different ratios of substrate-product concentrations. While K_M and K_i represent characteristic and constant enzyme parameters, V_{max} might differ significantly between time points and conditions under which (plant) metabolism is analysed. This might be due to a changing total enzyme amount or a changing temperature which affects reaction constants as described by the Arrhenius equation (Arrhenius, 1889). Altogether, this establishes a multi-parameter space for the estimation of a biochemical Jacobian matrix.

Its estimation from experimental data is challenged by the need for kinetic parameters which are sometimes not available or need to be acquired within laborious, difficult and error-prone experiments. To overcome experimental limitations by forward kinetic experiments, inverse estimation of ***J*** has been suggested based on covariance information of metabolite concentrations (Steuer et al., 2003). Here, fluctuation terms at a metabolic steady state are estimated from (co)variance information contained in metabolomics data. Applying this approach, regulatory hubs of metabolic networks can be identified without measuring enzyme kinetic parameters (Nägele et al., 2014; Sun & Weckwerth, 2012; Weckwerth, 2019; Wilson et al., 2020). However, experimental validation of predicted effects on enzymatic regulation is essential for such inverse estimations. Two main reasons why such inverse approximation might fail to correctly predict metabolic regulation are (i) strong deviation from steady state assumption due to significant internal and/or external perturbations, and (ii) misinterpretation of reasons for metabolite (co)variance, e.g., technical variance. Despite all experimental complications, a combined approach of inverse Jacobian matrix estimation and

experimental validation might support the analysis and understanding of complex metabolic network regulation (Wilson et al., 2020).

In summary, calculation and estimation of Jacobian matrices is a crucial element of system theory, and its application to biochemical networks seems mandatory to unravel complex regulatory principles. Entries of the Jacobian matrix are first-order partial derivatives of variable functions. As explained before, in a metabolic network context, it provides information about the effect of dynamics of one metabolite concentration on a specific metabolic function. Also, evaluation of eigenvalues of ***J*** indicates stability or instability of a metabolic system which provides important information about system behaviour at a considered steady state (Fürtauer & Nägele, 2016; Grimbs et al., 2007; Reznik & Segrè, 2010). Beyond, due to its tight regulation, many compounds of central (plant) primary metabolism are typically observed to be stabilized to a *quasi-steady state* without significant fluctuations of metabolite concentrations during a period of at least minutes, if not hours (Küstner et al., 2019; Nägele et al., 2012; Sulpice et al., 2014). Hence, although the Jacobian matrix is derived based on steady state assumptions, it is frequently applicable to study and predict dynamic system properties in complex networks within a certain period of experimental observations. For example, reconstruction of high-dimensional interaction Jacobian networks based on empirical time series data supported the identification of dynamical stability of a bacterial community (Chang et al., 2021). In another recent example, Jacobians were shown to support the study of control in networks with uncertain structure (Klickstein & Sorrentino, 2021) which highlights its central role for mathematical analysis in signalling and metabolic networks.

In the following paragraph, the Hessian matrix is introduced in context of metabolic regulation which comprises second-order partial derivatives. It is discussed in context of the provided example of invertase-catalysed sucrose cleavage.

Applying the Hessian matrix to study substrate-product-interactions on metabolic functions

The Hessian matrix of a function $f(x_1, x_2, x_3, \ldots, x_n)$ with n variables contains its second-order partial derivatives. Following Schwarz' theorem (also: Clairaut's theorem), it is an *n x n* symmetric matrix, i.e., the second-order partial derivatives satisfy identity regarding the order of differentiation (Eq. S10).

$$\frac{\partial}{\partial x_i}\left(\frac{\partial f}{\partial x_j}\right) = \frac{\partial}{\partial x_j}\left(\frac{\partial f}{\partial x_i}\right) = \frac{\partial^2 f}{\partial x_i \partial x_j}$$

(Eq. S10)

Considering metabolite concentrations as variables within metabolic functions of the given invertase reaction system, the Hessian matrices of *f(Suc)* calculates as follows (Eq. S11):

$$H_{f(Suc)}(Suc, Glc\ Frc) = \begin{pmatrix} h_{\mathrm{f(Suc)},11} & h_{\mathrm{f(Suc)},12} & h_{\mathrm{f(Suc)},13} \\ h_{\mathrm{f(Suc)},21} & h_{\mathrm{f(Suc)},22} & h_{\mathrm{f(Suc)},23} \\ h_{\mathrm{f(Suc)},31} & h_{\mathrm{f(Suc)},32} & h_{\mathrm{f(Suc)},33} \end{pmatrix} = \begin{pmatrix} \frac{\partial^2(r_{in} - r_{inv})}{\partial(Suc)^2} & \frac{\partial^2(r_{in} - r_{inv})}{\partial(Suc)\partial(Glc)} & \frac{\partial^2(r_{in} - r_{inv})}{\partial(Suc)\partial(Frc)} \\ \frac{\partial^2(r_{in} - r_{inv})}{\partial(Glc)\partial(Suc)} & \frac{\partial^2(r_{in} - r_{inv})}{\partial(Glc)^2} & \frac{\partial^2(r_{in} - r_{inv})}{\partial(Glc)\partial(Frc)} \\ \frac{\partial^2(r_{in} - r_{inv})}{\partial(Frc)\partial(Suc)} & \frac{\partial^2(r_{in} - r_{inv})}{\partial(Frc)\partial(Glc)} & \frac{\partial^2(r_{in} - r_{inv})}{\partial(Frc)^2} \end{pmatrix}$$

$(Eq. S11)$

Accordingly, Hessian matrices of metabolic functions of both reaction products, glucose and fructose, comprise second-order partial derivates of *f(Glc)* and *f(Frc)*, respectively (Eqs. S12 and S13).

$$H_{f(Glc)}(Glc, Suc, Frc) = \begin{pmatrix} h_{\mathrm{f(Glc)},11} & h_{\mathrm{f(Glc)},12} & h_{\mathrm{f(Glc)},13} \\ h_{\mathrm{f(Glc)},21} & h_{\mathrm{f(Glc)},22} & h_{\mathrm{f(Glc)},23} \\ h_{\mathrm{f(Glc)},31} & h_{\mathrm{f(Glc)},32} & h_{\mathrm{f(Glc)},33} \end{pmatrix} = \begin{pmatrix} \frac{\partial^2(r_{Inv} - r_{out,Glc})}{\partial(Glc)^2} & \frac{\partial^2(r_{Inv} - r_{out,Glc})}{\partial(Glc)\partial(Suc)} & \frac{\partial^2(r_{Inv} - r_{out,Glc})}{\partial(Glc)\partial(Frc)} \\ \frac{\partial^2(r_{Inv} - r_{out,Glc})}{\partial(Suc)\partial(Glc)} & \frac{\partial^2(r_{Inv} - r_{out,Glc})}{\partial(Suc)^2} & \frac{\partial^2(r_{Inv} - r_{out,Glc})}{\partial(Suc)\partial(Frc)} \\ \frac{\partial^2(r_{Inv} - r_{out,Glc})}{\partial(Frc)\partial(Glc)} & \frac{\partial^2(r_{Inv} - r_{out,Glc})}{\partial(Frc)\partial(Suc)} & \frac{\partial^2(r_{Inv} - r_{out,Glc})}{\partial(Frc)^2} \end{pmatrix}$$

$(Eq. S12)$

$$H_{f(Frc)}(Frc, Suc, Glc) = \begin{pmatrix} h_{\mathrm{f(Frc)},11} & h_{\mathrm{f(Frc)},12} & h_{\mathrm{f(Frc)},13} \\ h_{\mathrm{f(Frc)},21} & h_{\mathrm{f(Frc)},22} & h_{\mathrm{f(Frc)},23} \\ h_{\mathrm{f(Frc)},31} & h_{\mathrm{f(Frc)},32} & h_{\mathrm{f(Frc)},33} \end{pmatrix} = \begin{pmatrix} \frac{\partial^2(r_{Inv} - r_{out,Frc})}{\partial(Frc)^2} & \frac{\partial^2(r_{Inv} - r_{out,Frc})}{\partial(Frc)\partial(Suc)} & \frac{\partial^2(r_{Inv} - r_{out,Frc})}{\partial(Frc)\partial(Glc)} \\ \frac{\partial^2(r_{Inv} - r_{out,Frc})}{\partial(Suc)\partial(Frc)} & \frac{\partial^2(r_{Inv} - r_{out,Frc})}{\partial(Suc)^2} & \frac{\partial^2(r_{Inv} - r_{out,Frc})}{\partial(Suc)\partial(Glc)} \\ \frac{\partial^2(r_{Inv} - r_{out,Frc})}{\partial(Glc)\partial(Frc)} & \frac{\partial^2(r_{Inv} - r_{out,Frc})}{\partial(Glc)\partial(Suc)} & \frac{\partial^2(r_{Inv} - r_{out,Frc})}{\partial(Glc)^2} \end{pmatrix}$$

$(Eq. S13)$

The Hessian matrix provides information about simultaneous effects of a combination of concentration dynamics of substrates, products or other metabolic effectors on a metabolic function. For example, in an invertase reaction, entries $h_{\mathrm{f(Suc)},11} = \frac{\partial^2(r_{in}-r_{inv})}{\partial(Suc)^2}$, $h_{\mathrm{f(Suc)},12} = \frac{\partial^2(r_{in}-r_{inv})}{\partial(Suc)\partial(Glc)}$ and $h_{\mathrm{f(Suc)},13} = \frac{\partial^2(r_{in}-r_{inv})}{\partial(Suc)\partial(Frc)}$ provide information about how the metabolic

function of sucrose depends on dynamics of (i) sucrose ($h_{f(Suc),11}$), (ii) sucrose and glucose ($h_{f(Suc),12}$), and (iii) sucrose and fructose concentrations ($h_{f(Suc),13}$), respectively (Eqs. S14 -S16).

$$h_{\mathrm{f(Suc),11}} = \frac{\partial^2 (r_{in} - r_{inv})}{\partial (Suc)^2} = \frac{2K_{M,Suc}\, K_{i,Glc}\, K_{i,Frc}{}^2\, V_{max,inv}\, (Frc + K_{i,Frc})}{(Glc + K_{i,Glc})(K_{M,Suc}\, Frc + K_{M,Suc}\, K_{i,Frc} + K_{i,Frc} Suc)^3}$$

(Eq. S14)

$$h_{\mathrm{f(Suc),12}} = \frac{\partial^2 (r_{in} - r_{inv})}{\partial (Suc) \partial (Glc)} = \frac{K_{M,Suc} K_{i,Glc} K_{i,Frc} V_{max,inv} (Frc + K_{i,Frc})}{(Glc + K_{i,Glc})^2 (K_{M,Suc}\, Frc + K_{M,Suc}\, K_{i,Frc} + K_{i,Frc} Suc)^2}$$

(Eq. S15)

$$h_{\mathrm{f(Suc),13}} = \frac{\partial^2 (r_{in} - r_{inv})}{\partial (Suc) \partial (Frc)} = \frac{K_{M,Suc} K_{i,Glc} K_{i,Frc} V_{max,inv} \left(K_{M,Suc}\, Frc + K_{M,Suc} K_{i,Frc} - K_{i,Frc}\, Suc\right)}{(Glc + K_{i,Glc})\, (K_{M,Suc}\, Frc + K_{M,Suc}\, K_{i,Frc} + K_{i,Frc} Suc)^3}$$

(Eq. S16)

Competitive (Frc) and non-competitive inhibitors (Glc) differentially shape and affect the metabolic function of the reaction substrate (Suc) which is expressed in Hessian terms by different positions and exponents in nominators and denominators. While differential regulatory impact of different types of inhibitors on enzyme kinetics becomes evident from experimental enzyme kinetic analysis using purified enzymes, its interpretation in context of metabolic functions remains difficult. In this context, Jacobian and Hessian matrices provide important insight because they describe dynamics of metabolic functions with respect to a certain variable, e.g., a metabolic inhibitor.

Supplemental references

Arrhenius, S. (1889). Über die Reaktionsgeschwindigkeit bei der Inversion von Rohrzucker durch Säuren. *Zeitschrift für physikalische Chemie*, *4*(1), 226-248.

Chang, C.-W., Miki, T., Ushio, M., Ke, P.-J., Lu, H.-P., Shiah, F.-K., & Hsieh, C.-h. (2021). Reconstructing large interaction networks from empirical time series data. *Ecology Letters*, *24*(12), 2763-2774. https://doi.org/https://doi.org/10.1111/ele.13897

Doehlert, D. C., & Huber, S. C. (1983). Spinach leaf sucrose phosphate synthase. *FEBS Letters*, *153*(2), 293-297. https://doi.org/10.1016/0014-5793(83)80627-9

Fürtauer, L., & Nägele, T. (2016). Approximating the stabilization of cellular metabolism by compartmentalization. *Theory Biosci*, *135*(1-2), 73-87. https://doi.org/10.1007/s12064-016-0225-y

Granot, D., Kelly, G., Stein, O., & David-Schwartz, R. (2014). Substantial roles of hexokinase and fructokinase in the effects of sugars on plant physiology and development. *Journal of Experimental Botany*, *65*(3), 809-819. https://doi.org/10.1093/jxb/ert400

Grimbs, S., Selbig, J., Bulik, S., Holzhutter, H. G., & Steuer, R. (2007). The stability and robustness of metabolic states: identifying stabilizing sites in metabolic networks. *Mol Syst Biol*, *3*, 146. https://doi.org/10.1038/msb4100186

Heinrich, R., & Rapoport, T. A. (1974). A linear steady-state treatment of enzymatic chains. General properties, control and effector strength. *Eur J Biochem*, *42*(1), 89-95. http://www.ncbi.nlm.nih.gov/pubmed/4830198

Klickstein, I., & Sorrentino, F. (2021). Controlling network ensembles. *Nat Commun*, *12*(1), 1884. https://doi.org/10.1038/s41467-021-22172-6

Klipp, E., Liebermeister, W., Wierling, C., & Kowald, A. (2016). *Systems biology: a textbook*. John Wiley & Sons.

Küstner, L., Nägele, T., & Heyer, A. G. (2019). Mathematical modeling of diurnal patterns of carbon allocation to shoot and root in Arabidopsis thaliana. *npj Systems Biology and Applications*, *5*(1), 4. https://doi.org/10.1038/s41540-018-0080-1

Nägele, T., Mair, A., Sun, X., Fragner, L., Teige, M., & Weckwerth, W. (2014). Solving the differential biochemical Jacobian from metabolomics covariance data. *PLoS ONE*, *9*(4), e92299. https://doi.org/10.1371/journal.pone.0092299

Nägele, T., Stutz, S., Hörmiller, II, & Heyer, A. G. (2012). Identification of a metabolic bottleneck for cold acclimation in Arabidopsis thaliana. *Plant J*, *72*(1), 102-114. https://doi.org/10.1111/j.1365-313X.2012.05064.x

Reznik, E., & Segrè, D. (2010). On the stability of metabolic cycles. *J Theor Biol*, *266*(4), 536-549. https://doi.org/10.1016/j.jtbi.2010.07.023

Steuer, R., Kurths, J., Fiehn, O., & Weckwerth, W. (2003). Observing and interpreting correlations in metabolomic networks [Evaluation Studies

Validation Studies]. *Bioinformatics*, *19*(8), 1019-1026. https://doi.org/10.1093/bioinformatics/btg120

Stitt, M., Wirtz, W., & Heldt, H. W. (1983). Regulation of Sucrose Synthesis by Cytoplasmic Fructosebisphosphatase and Sucrose Phosphate Synthase during Photosynthesis in Varying Light and Carbon Dioxide. *Plant Physiol*, *72*(3), 767-774. http://www.ncbi.nlm.nih.gov/pubmed/16663083

Sulpice, R., Flis, A., Ivakov, A. A., Apelt, F., Krohn, N., Encke, B., Abel, C., Feil, R., Lunn, J. E., & Stitt, M. (2014). Arabidopsis Coordinates the Diurnal Regulation of Carbon Allocation and Growth across a Wide Range of Photoperiods. *Molecular Plant*, *7*(1), 137-155. https://doi.org/http://dx.doi.org/10.1093/mp/sst127

Sun, X. L., & Weckwerth, W. (2012). COVAIN: a toolbox for uni- and multivariate statistics, time-series and correlation network analysis and inverse estimation of the differential Jacobian from metabolomics covariance data. *Metabolomics*, *8*(1), S81-S93. https://doi.org/Doi 10.1007/S11306-012-0399-3

Volkert, K., Debast, S., Voll, L. M., Voll, H., Schießl, I., Hofmann, J., Schneider, S., & Börnke, F. (2014). Loss of the two major leaf isoforms of sucrose-phosphate synthase in Arabidopsis thaliana limits sucrose synthesis and nocturnal starch degradation but does not alter carbon partitioning during photosynthesis. *Journal of Experimental Botany*, *65*(18), 5217-5229. https://doi.org/10.1093/jxb/eru282

Wan, H., Wu, L., Yang, Y., Zhou, G., & Ruan, Y. L. (2018). Evolution of Sucrose Metabolism: The Dichotomy of Invertases and Beyond. *Trends Plant Sci*, *23*(2), 163-177. https://doi.org/10.1016/j.tplants.2017.11.001

Weckwerth, W. (2019). Toward a Unification of System-Theoretical Principles in Biology and Ecology—The Stochastic Lyapunov Matrix Equation and Its Inverse Application [Hypothesis and Theory]. *Frontiers in Applied Mathematics and Statistics*, *5*(29). https://doi.org/10.3389/fams.2019.00029

Wilson, J. L., Nagele, T., Linke, M., Demel, F., Fritsch, S. D., Mayr, H. K., Cai, Z., Katholnig, K., Sun, X., Fragner, L., Miller, A., Haschemi, A., Popa, A., Bergthaler, A., Hengstschlager, M., Weichhart, T., & Weckwerth, W. (2020). Inverse Data-Driven Modeling and Multiomics Analysis Reveals Phgdh as a Metabolic Checkpoint of Macrophage Polarization and Proliferation. *Cell Rep*, *30*(5), 1542-1552 e1547. https://doi.org/10.1016/j.celrep.2020.01.011
